# Supplementary material for: Weaker plant-enemy interactions decrease tree seedling diversity with edge-effects in a fragmented tropical forest
Source: Nat Commun. 2018 Oct 30;9:4523. doi: 10.1038/s41467-018-06997-2 (PMC6207651; doi:10.1038/s41467-018-06997-2)
Supplement: Supplementary file 1 — Supplementary information [file 41467_2018_6997_MOESM1_ESM.pdf]

SUPPLEMENTARY INFORMATION

Krishnadas et al., Weaker plant-enemy interactions decrease tree seedling diversity with edge-effects in a fragmented tropical forest

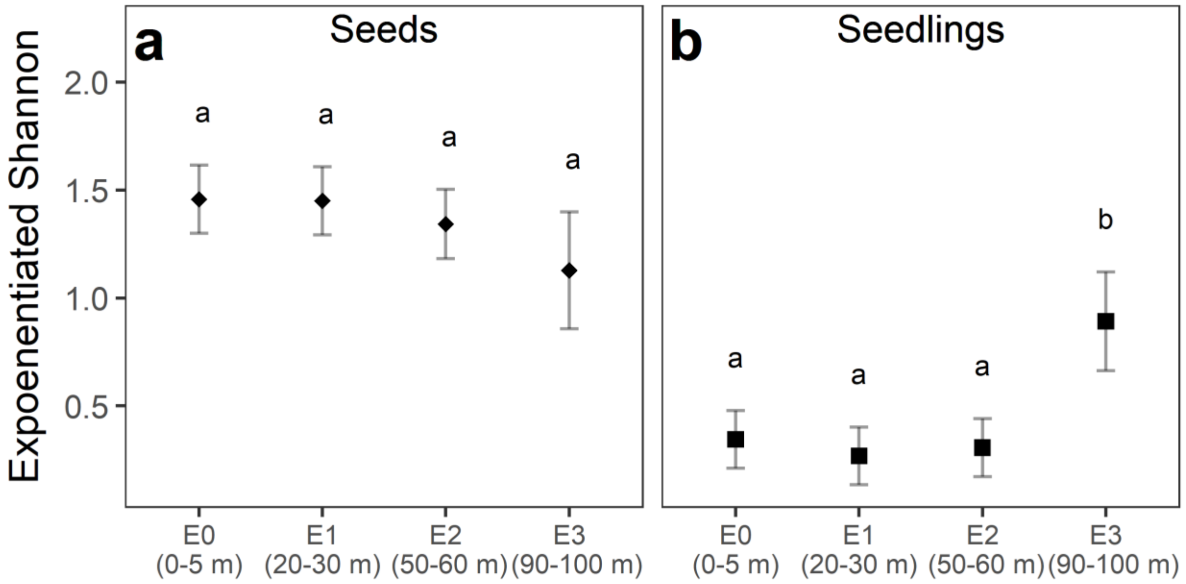

Supplementary Figure 1. Exponentiated Shannon diversity of seeds and seedlings at increasing distances from the forest edge. For a) seeds arriving in seed traps (N = 146) and b) seedlings that recruited into 1-m^2 control plots (N = 146), points represent mean values per edge distance and error bars show 95% Confidence Intervals. Larger values indicate higher diversity. E0 through E3 represent increasing distances from the forest edge. Letters denote significant pairwise differences among edge-distances (at p = 0.05), estimated using linear mixed-effects models.

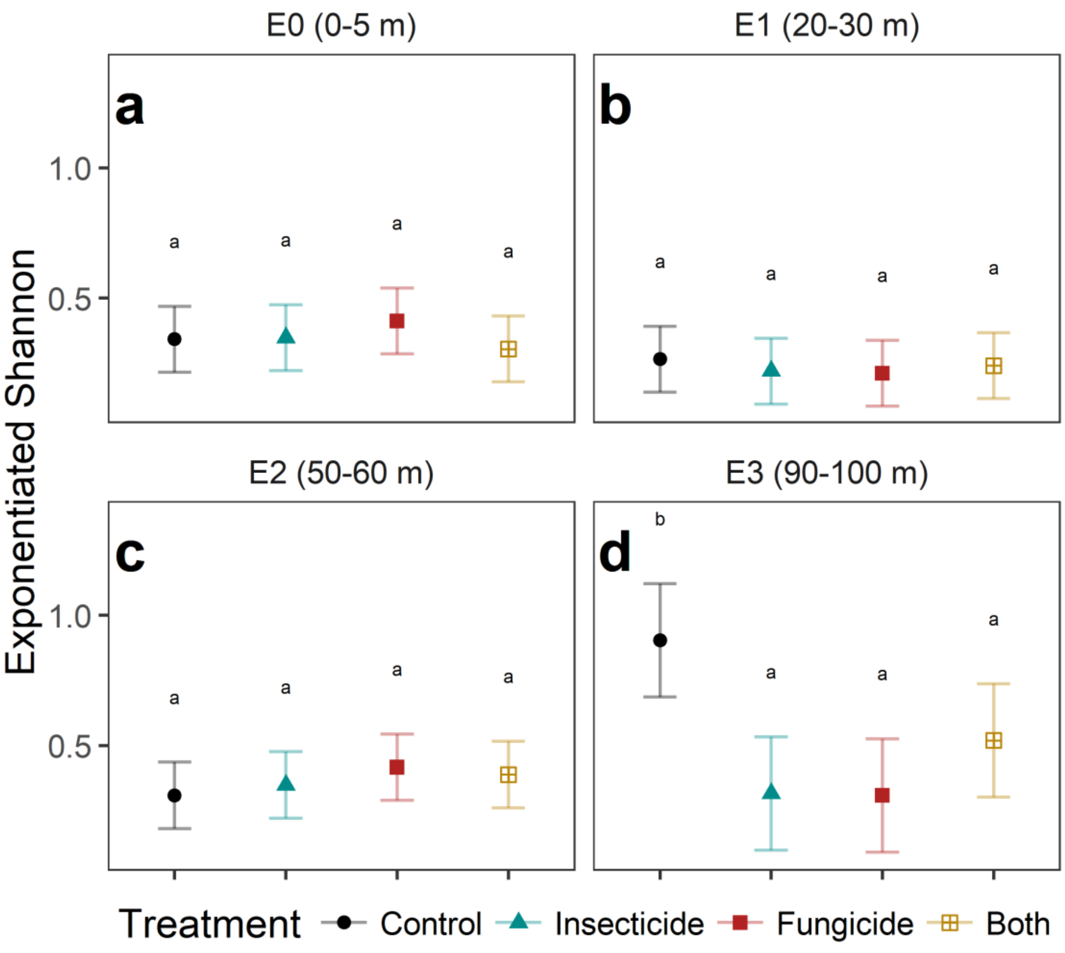

Supplementary Figure 2. Change in Exponentiated Shannon diversity with pesticide treatment at different distances from the forest edge. Sampling stations were established at: a) 0-5 m (E0) b) 20-30 m (E1) c) 50-60 m (E2) and d) E3 (90-100 m). Points represent mean observed diversity of new seedlings recruiting into 1-m^2 plots where the different pesticide treatments were applied (N = 146 per treatment). Error bars show 95% Confidence Intervals for the means. Letters denote significant pairwise differences (at p = 0.05) among treatments within each edge-distance category, estimated using linear mixed-effects models.

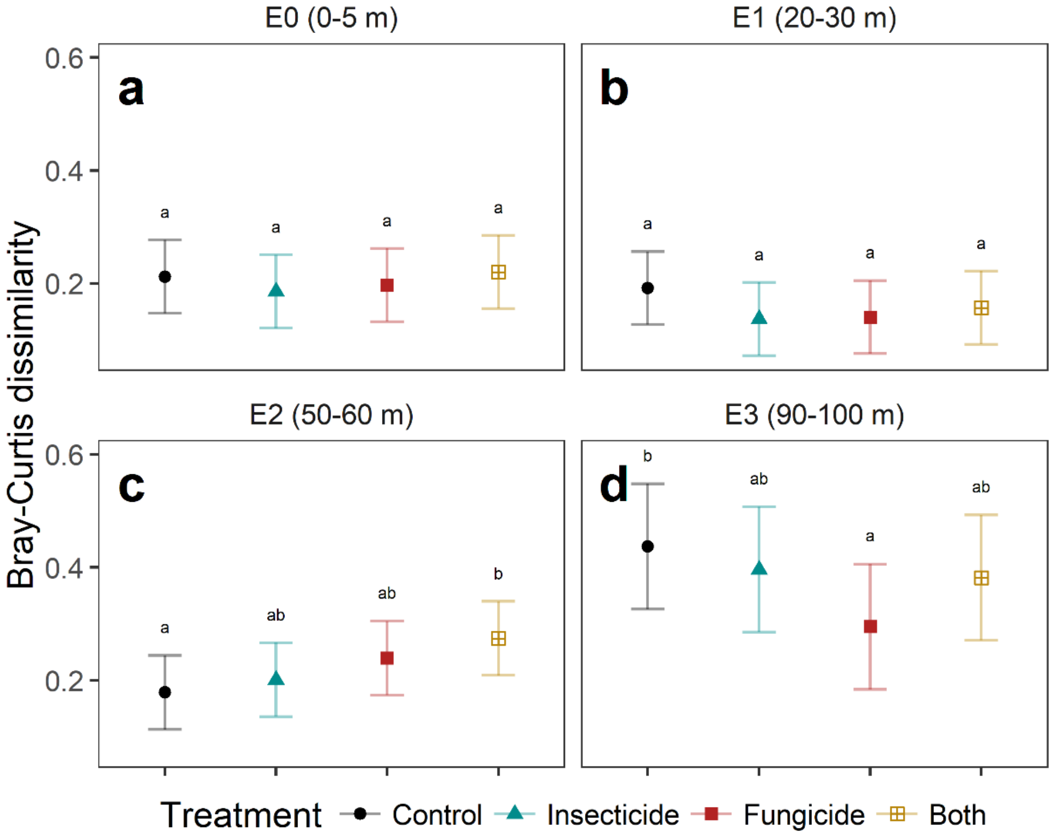

Supplementary Figure 3. Effect of pesticides on change in species composition between seed and seedling stages at different distances from the edge. Sampling stations were established at: a) E0: 0-5 m, b) E1: 20-30 m, c) E2: 50-60 m and d) E3: 90-100 m from the edge. Dissimilarity between seeds and seedlings, estimated using Bray-Curtis dissimilarity, was significantly higher at 90-100 m from forest edge (E3) compared to distances within 60 m of forest edge. In interior sites, dissimilarity between seeds and seedlings decreased significantly with fungicide application, but not with insecticide. Points represent mean observed dissimilarity (N = 146 per treatment). Error bars show 95% Confidence Intervals for the means. Letters denote significant pairwise differences among treatments within each edge-distance category (at $p = 0.05$), estimated using linear mixed-effects models.

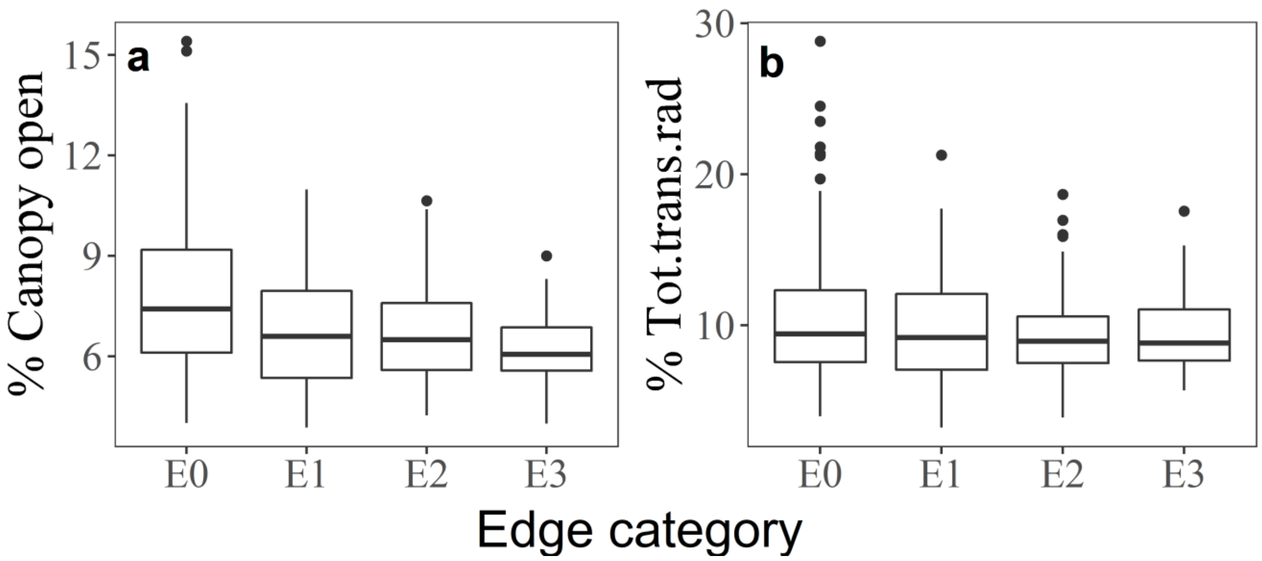

Supplementary Figure 4. Differences in canopy openness and light availability among edge-distances using hemispherical photos taken at the center of each 1-m^2 seedling plot. For each distance category (E0: 0-5 m, E1: 20-30 m, E2: 50-60 m, E3: 90-100 m from forest edge; N = 584): a) percent canopy openness and b) light availability measured as percent total transmitted radiation. Median canopy openness was significantly higher at E0 (Kruskal-Wallis test: chi-squared = 33.1, df = 3, p-value < 0.01) compared to all other distance categories that did not differ from each other (pairwise Wilcoxon rank-sum test). Median light availability did not differ among the distance categories (Kruskal-Wallis test: chi-squared = 2.6, df = 3, p-value = 0.46).

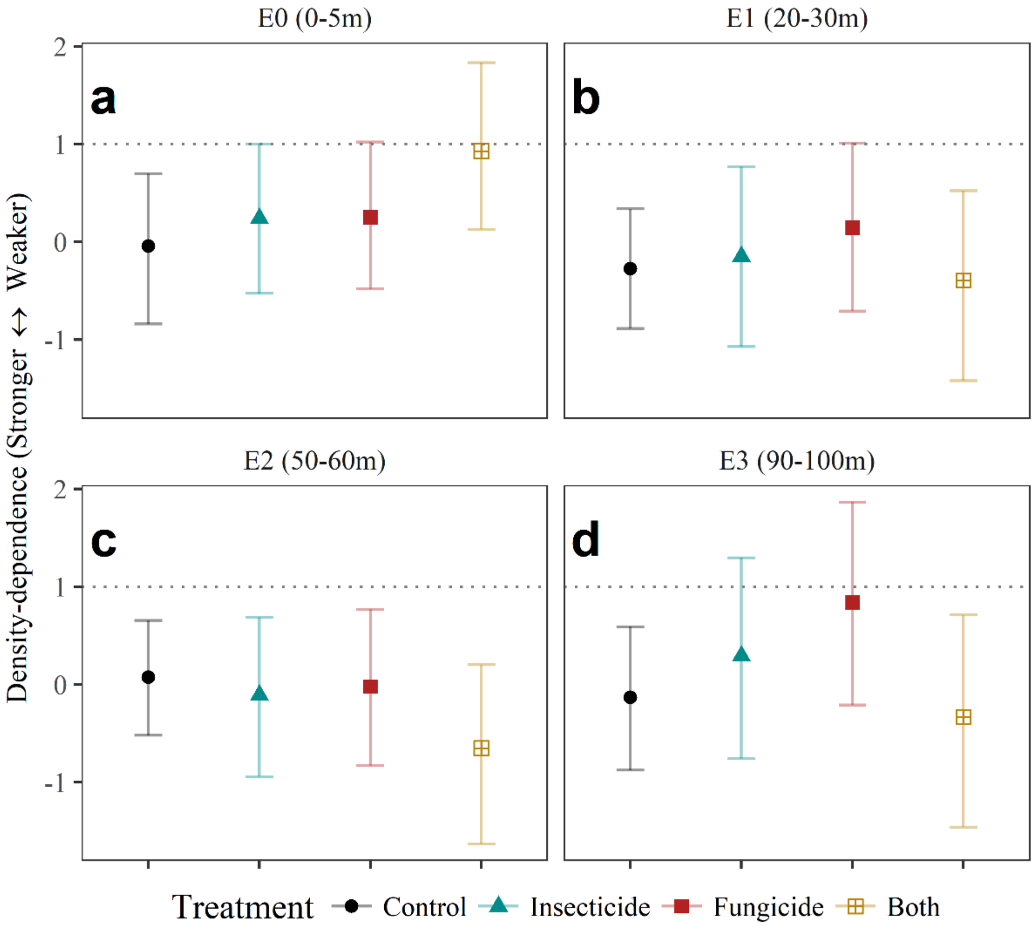

Supplementary Figure 5. For the subset of locations with all edge categories, change in strength of CNDD in relation to pesticide treatment at increasing distances from the forest edge. CNDD was estimated for: a) 0-5 m (E0) b) 20-30 m (E1) c) 50-60 m (E2) and d) E3 (90-100 m) (N = 45 per treatment). Points represent estimated strength of conspecific density dependence and bars provide 95% Bayesian Credible Intervals, estimated using generalized linear mixed-effects models in a hierarchical Bayesian framework. Dotted line represents no CNDD (slope=1). We estimated separate slopes per species and group-level intercepts per species and station (i.e. random slopes and intercepts, respectively).

Supplementary Table 1. Parameter estimates from generalized linear mixed-effects model testing variation in seedling abundance per 1-m^2 plot in relation to an interaction between distance from forest edge (E0, E1, E2, E3) and pesticide treatment (Insecticide, Fungicide, Both).

	**Estimate**	**SE**	**Z-value**	**P-value**
Intercept	1.14	0.18	6.48	0.00
E1 (20-30m)	-0.08	0.25	-0.30	0.76
E2 (50-60m)	0.01	0.25	0.04	0.97
E3 (100+m)	0.08	0.34	0.25	0.81
Insecticide	-0.12	0.17	-0.68	0.50
Fungicide	-0.12	0.17	-0.70	0.49
Both	0.14	0.16	0.87	0.39
E1 : Insecticide	0.18	0.24	0.73	0.46
E2 : Insecticide	0.06	0.24	0.25	0.80
E3 : Insecticide	-0.26	0.34	-0.78	0.44
E1 : Fungicide	0.01	0.24	0.06	0.96
E2 : Fungicide	0.27	0.24	1.14	0.25
E3 : Fungicide	-0.43	0.35	-1.24	0.21
E1 : Both	-0.14	0.23	-0.58	0.56
E2 : Both	0.18	0.23	0.76	0.44
E3 : Both	-0.37	0.33	-1.13	0.26

Supplementary Table 2. Parameter estimates from linear mixed-effects model testing variation in seedling abundance per 1-m^2 plot in relation to an interaction between light availability (% total transmitted radiation) and distance from forest edge (E0, E1, E2, E3).

	Estimate	SE	Z-value	P-value
(Intercept)	1.09	0.44	2.44	0.02
E1 (20-30m)	0.49	0.59	0.82	0.41
E2 (50-60m)	-0.15	0.66	-0.23	0.82
E3 (100+m)	0.41	1.05	0.39	0.70
Light	0.00	0.04	-0.09	0.93
E1 : Light	-0.04	0.05	-0.85	0.40
E2 : Light	0.02	0.07	0.30	0.76
E3 : Light	-0.03	0.09	-0.32	0.75

Supplementary Table 3. Community-wide changes in strength of conspecific negative density dependence (CNDD) during seed-to-seedling transition in relation to a) edge-distance x pesticide interactions and b) light availability (% total transmitted radiation) x pesticide interactions. Parameters were estimated using generalized linear mixed-effects models in a hierarchical Bayesian framework to incorporate measurement error due to spatial mismatch in seed fall into traps and seedling recruitment in adjacent plots. Separate slopes were estimated for each species and group-level errors for the intercept were estimated per species and station.

	**Estimate**	**SE**	**Lower CI**	**Upper CI**
**a. Distance from forest edge**				
Intercept	-0.99	0.17	-1.33	-0.66
E1 (20-30m)	-0.07	0.25	-0.55	0.41
E2 (50-60m)	-0.02	0.24	-0.49	0.46
E3 (100+m)	0.39	0.31	-0.18	1.02
Insecticide	-0.49	0.27	-1.00	0.03
Fungicide	-0.21	0.26	-0.72	0.31
Both	-0.77	0.28	-1.32	-0.22
E1 : Insecticide	-0.18	0.39	-0.96	0.59
E2 : Insecticide	0.04	0.37	-0.68	0.74
E3 : Insecticide	-0.55	0.50	-1.53	0.38
E1 : Fungicide	-0.80	0.40	-1.55	-0.04
E2 : Fungicide	-0.07	0.35	-0.76	0.62
E3 : Fungicide	-1.53	0.57	-2.69	-0.45
E1 : Both	0.32	0.38	-0.44	1.08

E2 : Both	0.54	0.37	-0.16	1.27
E3 : Both	-0.14	0.48	-1.08	0.82
CNDD	0.16	0.22	-0.27	0.60
CNDD : E1	0.01	0.13	-0.25	0.27
CNDD : E2	-0.05	0.13	-0.31	0.21
CNDD : E3	-0.36	0.28	-0.89	0.20
CNDD : Insecticide	0.11	0.14	-0.17	0.39
CNDD : Fungicide	0.05	0.15	-0.24	0.33
CNDD : Both	0.37	0.14	0.09	0.66
CNDD : E1 : Insecticide	0.13	0.20	-0.26	0.51
CNDD : E2 : Insecticide	0.00	0.19	-0.37	0.37
CNDD : E3 : Insecticide	**0.38**	**0.38**	**-0.34**	**1.10**
CNDD : E1 : Fungicide	0.24	0.20	-0.15	0.63
CNDD : E2: Fungicide	0.18	0.19	-0.18	0.55
CNDD : E3 : Fungicide	**0.96**	**0.41**	**0.16**	**1.78**
CNDD : E1 : Both	-0.21	0.19	-0.60	0.17
CNDD : E2 : Both	-0.08	0.19	-0.47	0.30
CNDD : E3 : Both	0.19	0.37	-0.55	0.91
**b. Light availability**				
Intercept	-1.23	0.34	-1.89	-0.55
Light	-0.03	0.03	-0.10	0.04
Insecticide	-0.92	0.57	-2.03	0.17
Fungicide	-0.65	0.53	-1.63	0.41
Both	-1.18	0.54	-2.22	-0.13
Light : Insecticide	0.02	0.06	-0.09	0.13

Light : Fungicide	0.00	0.06	-0.11	0.10
Light : Both	0.06	0.05	-0.05	0.15
CNDD	0.38	0.54	-0.66	1.45
CNDD : Light	0.05	0.05	-0.04	0.14
CNDD: Insecticide	1.31	0.76	-0.14	2.82
CNDD: Fungicide	0.45	0.76	-1.09	1.92
CNDD: Both	1.74	0.59	0.55	2.88
CNDD: Light : Insecticide	-0.09	0.08	-0.24	0.06
CNDD: Light : Fungicide	0.01	0.09	-0.15	0.18
CNDD: Light : Both	-0.11	0.06	-0.23	0.00

Supplementary Table 4. Parameter estimates from generalized linear mixed-effects model with binomial errors to test variation in recruitment rates per 1-m^2 plot in relation to an interaction between distance from forest edge (E0, E1, E2, E3) and pesticide treatment. Species and station were included as random effects to account for interspecific differences and spatial location.

	**Estimate**	**SE**	**Z-value**	**P-value**
Intercept	-2.30	0.35	-7.74	0.00
E1 (20-30m)	-0.44	0.24	-1.28	0.20
E2 (50-60m)	**-0.45**	**0.24**	**-3.57**	**0.00**
E3 (90-100m)	1.19	0.34	1.59	0.11
Insecticide	**-0.80**	**0.11**	**-7.96**	**0.00**
Fungicide	**-0.80**	**0.11**	**-8.02**	**0.00**
Both	0.04	0.10	0.44	0.66
E1 : Insecticide	**0.56**	**0.16**	**4.22**	**0.00**
E2 : Insecticide	**0.69**	**0.15**	**5.13**	**0.00**
E3 : Insecticide	-0.05	0.26	0.01	0.99
E1 : Fungicide	**0.55**	**0.16**	**4.19**	**0.00**
E2 : Fungicide	**1.54**	**0.15**	**11.59**	**0.00**
E3 : Fungicide	0.09	0.25	0.55	0.58
E1 : Both	**-0.37**	**0.15**	**-2.51**	**0.01**
E2 : Both	**1.01**	**0.13**	**7.51**	**0.00**
E3 : Both	**-0.73**	**0.25**	**-2.97**	**0.00**

Supplementary Table 5. Parameter estimates from linear mixed-effects model testing variation in seedling diversity per 1-m^2 plot in relation to an interaction between light availability (% total transmitted radiation) and pesticide treatment (Insecticide, Fungicide, Both). Here we present results for Inverse Simpson and patterns were similar for exponentiated Shannon.

	**Estimate**	**SE**	**Z-value**	**P-value**
Intercept	1.44	0.23	6.14	0.00
Seed diversity	0.00	0.02	-0.12	0.91
% Trans. tot.	-0.01	0.02	-0.53	0.59
Insecticide	-0.31	0.32	-0.95	0.34
Fungicide	-0.21	0.29	-0.71	0.48
Both	0.04	0.31	0.17	0.86
% Trans. tot.: Insecticide	0.01	0.03	0.37	0.70
% Trans. tot.: Fungicide	0.01	0.03	0.18	0.85
% Trans. tot.: Both	0.00	0.03	-0.11	0.91

Supplementary Table 6. Parameter estimates from linear mixed-effects model testing variation in seedling diversity per 1-m^2 plot in relation to an interaction between percent canopy openness (proxy for moisture and humidity) and pesticide treatment (Insecticide, Fungicide, Both). Here we present results for Inverse Simpson and patterns were similar for exponentiated Shannon.

	**Estimate**	**SE**	**Z-value**	**P-value**
Intercept	1.13	0.31	3.75	0.03
Seed diversity	0.00	0.03	0.00	0.99
% Canopy open	0.03	0.04	0.88	0.37
Insecticide	-0.19	0.41	-0.46	0.65
Fungicide	-0.02	0.40	-0.04	0.97
Both	0.20	0.38	0.54	0.59
% Canopy open: Insecticide	0.00	0.06	0.01	0.99
% Canopy open: Fungicide	-0.02	0.05	-0.34	0.73
% Canopy open: Both	-0.04	0.05	-0.79	0.43

Supplementary Table 7. Seedling diversity in relation to edge x pesticide interactions for the 15 replicates with all distance categories (i.e. including the interior-most treatment E3: 90-100 m). Results are similar to analysis conducted with all locations (Fig. 3). Here we present results for Inverse Simpson and patterns were similar for exponentiated Shannon.

	**Estimate**	**SE**	**Z-value**	**P-value**
Intercept (E0, 0-5 m)	0.98	0.28	3.50	0.00
Seed diversity	-0.05	0.04	-1.33	0.18
E1 (20-30 m)	0.22	0.32	0.67	0.50
E2 (50-60 m)	0.39	0.32	1.20	0.23
E3 (90-100 m)	**1.53**	**0.33**	**4.68**	**0.00**
Insecticide	0.23	0.29	0.78	0.44
Fungicide	0.25	0.29	0.87	0.38
Both	0.06	0.29	0.22	0.82
E1 : Insecticide	-0.52	0.41	-1.26	0.21
E2 : Insecticide	-0.11	0.41	-0.26	0.80
E3 : Insecticide	**-1.17**	**0.41**	**-2.84**	**0.00**
E1 : Fungicide	-0.39	0.41	-0.94	0.35
E2 : Fungicide	-0.01	0.41	-0.02	0.98
E3 : Fungicide	**-1.49**	**0.41**	**-3.61**	**0.00**
E1 : Both	-0.22	0.41	-0.53	0.60
E2 : Both	0.16	0.41	0.38	0.70
E3 : Both	-0.68	0.41	-1.65	0.10
